# Gastroprotective effects of *Caragana ambigua* stocks on ethanol‐induced gastric ulcer in rats supported by LC–MS/MS characterization of formononetin and biochanin A

**DOI:** 10.1002/jsfa.12064

**Published:** 2022-07-11

**Authors:** Faryal Rubab, Hira Ijaz, Shah Hussain, Ans Munir, Stefan Stuppner, Thomas Jakschitz, Günther K Bonn, Saiqa Ishtiaq

**Affiliations:** ^1^ Punjab University College of Pharmacy University of the Punjab Lahore Pakistan; ^2^ ADSI–Austrian Drug Screening Institute GmbH Innsbruck Austria; ^3^ Department of Chemistry GC University Lahore Pakistan; ^4^ Institute of Analytical Chemistry and Radiochemistry, CCB‐Center for Chemistry and Biomedicine Leopold‐Franzens University Innsbruck Austria

**Keywords:** *Caragana ambigua*, Fabaceae, gastric ulcer, formononetin, biochanin A

## Abstract

**BACKGROUND:**

*Caragana ambigua* has been the part of the dietary routines of the regional people in south‐west Pakistan and has traditionally been used for the treatment of diabetes there. There is an increased production of reactive oxygen species in diabetics, leading to gastrointestinal disorders. Natural antioxidants exhibit gastroprotective effects owing to their free‐radical scavenging action. *C. ambigua* possesses appreciable phenolic and flavonoid content; thus, it has the potential to protect against gastrointestinal disorders (e.g. gastric ulcer).

**RESULTS:**

This study reports the anti‐ulcer potential of *C. ambigua*. Four different fractions (chloroform, ethyl acetate, butanol, and aqueous) of plant were compared against omeprazole. Ulcer index, ulcer inhibition percentage, gastric pH and volume, total acidity, gastric protein, gastric wall mucus, and histopathology of gastric walls of rats were assessed. All fractions exhibited a reduction in ulcer index and promotion of percentage of ulcer inhibition compared with the ulcer control group. Furthermore, the fractions revealed a significant (*P* < 0.001) diminution in gastric volume and total acidity with an increase in pH. Among the fractions investigated, the chloroform fraction unveiled the most promising anti‐ulcer activity, which is comparable to omeprazole. Liquid chromatography–tandem mass spectrometry screening of fractions revealed the presence of formononetin and biochanin A (isoflavones reported to have anti‐ulcer properties) in the chloroform fraction.

**CONCLUSION:**

This study establishes that *C. ambigua* possesses significant potential in reducing gastric ulcer progression. Formononetin and biochanin A are chiefly responsible for the stated bioactivity due to the fact that these compounds were solely present in the chloroform fraction. © 2022 The Authors. *Journal of The Science of Food and Agriculture* published by John Wiley & Sons Ltd on behalf of Society of Chemical Industry.

## INTRODUCTION

Gastric ulcer (GU) is developed due to persistent erosions of mucosal membrane with ensuing hemorrhage of the gastric mucosa.^1^ Disturbance of equilibrium between gastric acid production and mucus turnover rate plays a foremost role in GU development.^2^ These physiological changes result due to multiple factors, including infection caused by *Helicobacter pylori*, non‐steroidal anti‐inflammatory drugs, stress, smoking, alcohol, and dietary habits.^3^ All these factors produce oxidative stress that plays a critical part in the progression of the ulcer. At the site of injury, neutrophils and other inflammatory mediators accumulate in response to reactive oxygen species (ROS). These ROS impair the cellular functions by promoting damage to the mucosal membrane via destroying the cellular organelles, like lysosomes, mitochondria, and the nucleus.^4^ GU can be cured either by inhibiting acid production or boosting mucus turnover rate via therapeutic agents like histamine receptor antagonists, antacids, and proton‐pump inhibitors.^5^ Nevertheless, these therapeutics cause severe adverse effects, such as hyperplasia in enterochromaffin‐like cells, which can lead to ulcer relapse as well as induction of stomach cancer.^6^ Other related adverse effects are arrhythmia, hypersensitivity, impotency, and hematopoietic disturbances.^7^ Concisely, the limitations of the aforementioned commercial therapeutics strengthen the urge to investigate other treatment options with potentially less or no side effects, having more efficiency and patient compliance. Therefore, natural products are making progress in the pharma industry by introducing potential sources of bioactive compounds.^8^ In conventional Chinese medicines, plants belonging to the genus *Caragana* have been used to address rheumatism, bruises, contused wounds, and hemorrhages. Namely, *Caragana jubata* and *Caragana frutex* have been used in the treatment of skin ulcers, carbuncles, and abscesses. Moreover, roots of *Caragana rosea* have been used in the treatment of stomach deficiencies and to invigorate the stomach.^9^ A wide range of biological and chemical aspects of different species of *Caragana* have been reported; however, some of the species remain unexplored. One such species that has not been fully explored in terms of its chemical ingredients and biological potential is *Caragana ambigua*. Traditionally, juice obtained from the whole plant of *C. ambigua* has been used to treat diabetes mellitus in south‐west Pakistan.^10^ It has also been used as fodder by the native community of Baluchistan (a major province of Pakistan).^11^, ^12^

This species encompasses high phenolic contents, along with alkaloids, saponins, and tannins.^13^ *C. ambigua* seeds contain a unique peptide, called *C. ambigua* seed peptide, that has pronounced antioxidant potential. This exclusive peptide has the ability to slow down lipid oxidation rates and protect a variety of polyunsaturated fatty acids from auto‐oxidation.^14^ More recent research also validates the appreciable phenolic and flavonoid content of *C. ambigua* along with its significant antioxidant potential.^15^ Antioxidants from food and drugs play an important part in inhibiting the progression of GU owing to their ability to neutralize the toxic effects of ROS, and presumably *C. ambigua* may serve this purpose. Literature has also provided scientific data regarding the anti‐diabetic potential of *C. ambigua*.^15^, ^16^ Diabetes is characterized by hyperglycemia, depletion of antioxidants, and altered lipid metabolism; therefore, diabetic patients have a significantly higher prevalence of GU. Moreover, GU that develop in diabetic subjects are more severe and are often associated with complications.^17^ Therefore, in addition to being useful in the treatment of diabetes, *C. ambigua* may provide additional benefits of anti‐ulcer potential to the diabetic patients. Moreover, *Caragana spinosa* has been found to possess anti‐ulcer potential.^18^ Keeping in view the pharmacological relevance of the genus, the extensive use of *C. ambigua* as a fodder, the usefulness of *C. ambigua* in the treatment of diabetes mellitus, and the reported significant antioxidant potential, this study was intended to investigate the anti‐ulcer potential of *C. ambigua*. For this purpose, *in vivo* biological studies of different fractions from *C. ambigua* were carried out by developing ulcer model in rats. Moreover, Liquid chromatography (LC)–tandem mass spectrometry (MS/MS) screening of the *C. ambigua* fractions could afford the characterization of formononetin and biochanin A (isoflavones known to have anti‐ulcer potential). To the best of our knowledge, this study is the first instance of the determination of the anti‐ulcer potential of *C. ambigua* and reports for the first‐time the presence of the stated isoflavones in this plant.

## MATERIALS AND METHODS

### Plant collection

*C. ambigua* plant was collected in May 2016 from Ziarat, Pakistan. The voucher number GC.Herb.Bot.3446 was assigned and submitted to the Herbarium of GC University, Lahore, Pakistan.

#### Preparation of plant extracts and its fractions

Fresh aerial parts of plant were collected and dried in shade. Subsequently, 500 g of dried plant material was pulverized using a mechanical mill. The powdered sample was then subjected to maceration using methanol (2500 mL) as solvent for 7 days. The extract was then dried using rotary evaporation under reduced pressure. The residue obtained (i.e. crude methanolic extract, CA‐M), 66.92 g, was then suspended in water (50 mL) and partitioned with chloroform (250 mL), ethyl acetate (250 mL), *n*‐butanol (250 mL), and water. The *C. ambigua* chloroform (CA‐C), *C. ambigua* ethyl acetate (CA‐E), *C. ambigua n*‐butanol (CA‐B), and *C. ambigua* aqueous (CA‐Aq) fractions were dried using rotary evaporation and stored in a refigerator.^19^

#### Animals

Male and female albino rats (weighing 150–200 g) were used in the experiments. All animals were kept in a place with proper ventilation, temperature‐maintained 23 ± 2 °C and 12 h/12 h period of light and dark. Prior to the induction of ulcer, the animals were withheld from food for 24 h but had free access to water for 2 h.^20^ The rats were grouped and placed in different cages. The experiment was performed with the permission of the Bio‐ethical Committee from the Department of Zoology, University of The Punjab, Lahore, Pakistan (Ethical Approval ID: 1594), and the approval date was 22 November 2019.

#### Drugs and chemicals

Omeprazole was kindly provided by Surge Laboratories (Pvt.) Ltd (Chah Chandu, Sheikhupura, Pakistan) and was used as reference drug. Normal saline was from Otsuka Pakistan Ltd (Lasbella, Pakistan), whereas absolute ethanol, bovine serum albumin and alcian blue 8 GX were from Sigma–Aldrich (St Louis, MO, USA). Sodium carbonate, sodium hydroxide (NaOH), sucrose, diethyl ether, and magnesium chloride were from BDH Chemicals Ltd (Poole, UK). Copper sulfate pentahydrate, sodium acetate, Tris buffer hydrochloride, and Folin–Ciocalteu reagent were from Central Drug House (New Delhi, India). Disposable polytetrafluoroethylene (PTFE) syringe filters (pore size 0.2 μm, hydrophobic, 30 mm) were from Carl Roth GmbH+Co. KG (Karlsruhe, Germany). All the reagents were freshly prepared and of pharmaceutical grade.

### Acute toxicity studies

Acute oral toxicity testing was performed to determine the safe dose of different fractions of *C. ambigua*. Rats were randomly divided into five groups that received vehicle orally (distilled water, 5 mL kg^−1^ body weight (BW)), CA‐C (2000 mg kg^−1^ BW), CA‐E (2000 mg kg^−1^ BW), CA‐B (2000 mg kg^−1^ BW), and CA‐Aq (2000 mg kg^−1^ BW). Animals were continuously monitored for 4 h after dosing for any behavioral changes or clinical manifestations of toxicity. Mortality was recorded, if any. The administered rats were followed up for 14 days. On the 15th day, blood samples were taken and animals were sacrificed. The liver and kidney were taken for histopathological analysis.^21^

### Ethanol‐induced GU model

The animals were subjected to division into seven groups, with each group holding six rats. Animals were fasted for 24 h with free access to water until 2 h prior to experiment. The normal control group (group I) and the ulcer control group (group II) received distilled water in a single volume (1 mL per animal) orally. The positive control group (group III) was administered with an oral dose of omeprazole (20 mg kg^−1^ BW) in a single volume (1 mL per animal). Groups IV, V, VII, and VIII served as the experimental group and received oral doses of CA‐C, CA‐E, CA‐B, and CA‐Aq (300 mg kg^−1^ BW dissolved in water) in a single volume (1 mL per animal) respectively.^22^ After 30 min, all the animals of groups II–VII were administered ethanol for ulcer induction (1 mL per animal).^23^ After 1 h, all the animals were euthanized with an overdose of anesthetic drugs (i.e. ketamine and xylazine). Stomachs were removed and their contents were emptied and collected. Stomachs were opened along the greater curvature, washed with normal saline, and then placed on a white board for ulcer score calculation.^24^

#### Calculation of ulcer index

A magnifying glass was used to count the ulcers. Severity of ulcer was indicated in terms of scores as follows: 0 = normal coloration, 0.5 = red coloration, 1.0 = spot ulcer, 1.5 = hemorrhagic streaks, 2.0 = deep ulcer, and 3.0 = perforations.^25^

Ulcer index (UI) was calculated according to^26^

$$\mathrm{UI}=\left(\mathrm{UN}+\mathrm{US}+\mathrm{UP}\right)\;\times \;10^{-1}$$

where UN is the average number of ulcers per animal, US is the average of severity score, and UP is the percentage of animals with ulcer.

##### Percentage of ulcer protection

The following formula was used to evaluate the percentage protection:^26^

$$\text{Protection}\;\left(\%\right)=\frac{\mathrm{UI}\;\left(\text{Ulcer control}\right)-\mathrm{UI}\;\left(\text{Treated}\right)}{\mathrm{UI}\;\left(\text{Ulcer control}\right)}\;\times \;100$$

##### Histopathological evaluation

From each rat, gastric wall specimens were taken, fixed in formalin, processed, and then embedded in paraffin. Stomach sections were cut into 5 mm thicknesses and subjected to staining with eosin and hematoxylin for histopathological examination.^27^

##### Estimation of gastric volume and pH

Gastric contents were collected in Falcon tubes (10 mL) and centrifuged at 3000 × *g* for 10 min. Gastric juice volume and pH of supernatant were analyzed for each sample.^28^

##### Estimation of total gastric acidity

Using phenolphthalein as indicator, 1 mL of gastric juice was titrated against 0.1 mol L^−1^ NaOH until the end point denoting colorless to light pink was attained. The volume of NaOH used was noted and total acidity was measured as follows:^29^

$$\;\text{Total gastric acidity}=\frac{\left(\mathrm{Vol},.,\;,\text{of NaOH},\;,\times ,\;,\text{normality of NaOH}\right)}{0.1\;\times \;100\;\mathrm{mEq}\;L^{-1}}$$

##### Estimation of gastric wall protein

Protein estimation was achieved by following the method described by Lowry *et al*.^30^

##### Estimation of gastric mucus content

Glandular portions were removed from the stomachs of all animals, weighed, and immediately transferred to 0.1% alcian blue (in 0.16 mol L^−1^ sucrose solution buffered with 0.05 sodium acetate adjusted to pH 5). The excess dye was removed by rinsing with 0.25 mol L^−1^ sucrose solution at intervals of 15 and 45 min. The dye, which formed a complex with the gastric wall mucus, was extracted by using 10 mL of 0.5 mol L^−1^ magnesium chloride. With an equal volume of diethyl ether, 4 mL of the blue extract were shaken. The emulsion thus obtained was centrifuged and the absorbance of the aqueous layer was measured at 580 nm.^31^

### Ultra‐high performance LC coupled with ultraviolet and high‐resolution quadrupole time‐of‐flight MS/MS

A 100 mg sample of each dry fraction was dissolved in 100 mL ethanol and placed in an ultrasonic bath for 30 min. Subsequently, the fractions were filtered through 0.2 μm PTFE syringe filters. A 100‐fold dilution was performed for ultra‐high performance LC (UHPLC) coupled with ultraviolet (UV) and high‐resolution quadrupole time‐of‐flight (hr‐qTOF) MS/MS in full scan mode and 50‐fold dilution was performed for MS/MS analysis.

#### Instrumentation

A Thermo Scientific Dionex UltiMate 3000 coupled with a maxis Impact Ultra High Resolution TOF‐MS instrument from Bruker Daltonics (Bremen, Germany) was used for the analysis. An Agilent (Agilent Technologies, GmbH, Vienna, Austria) RRHD Zorbax C18 (2.1 x 100 mm, 1.8 μm) column was used for chromatographic separations. Acetonitrile (solution B) and 0.1% formic acid in water (solution A) was used as mobile phase. The flow rate was 0.4 mL min^−1^, the injection volume was 5 μL, and the column oven was set at 45 °C. The LC elute was directly infused into the mass spectrometer with mass scanning from 80 to 1500 *m*/*z* and spectra rate 4 Hz, using electrospray ionization in positive mode. The UV spectra were recorded at 280 nm. Compass Data Analysis 4.2 from Bruker® was used for the interpretation of the mass signals. Each sample was measured in the form of technical triplicates. The LC gradient consisted of the following linear steps (min/B %): 0/3, 15/50, 18/80, 19/100, 21/100, 23/3. The MS parameters were as follows: nitrogen nebulizer gas pressure, 3 bar; drying gas flow rate, 12 L min^−1^; end plate offset, 500 V; capillary voltage, +4500 V; dry temperature, 200 °C; funnel 1 radiofrequency (RF) and funnel 2 RF, 300 V_pp_; collision‐induced dissociation energy, 0 eV; hexapole RF, 50 V_pp_; quadrupole ion energy, 5 eV; and low mass filtering at 50 *m*/*z*. Collision cell parameters were adjusted as follows: collision energy, 3 eV; collision RF, 500 V_pp_; transfer time 50 μs; and pre‐pulse storage was at 6 μs. For data analysis, Data Analysis 4.2 (Bruker Daltonics) software was used.

## STATISTICAL ANALYSIS

The results are displayed as mean plus/minus standard error of the mean. Statistically, the data were analyzed by applying one‐way analysis of variance, which was followed by Dunnett's test, and *P*‐values <0.05 were considered significant.

## RESULTS

### Acute toxicity studies

The results revealed that all the fractions of *C. ambigua*, even after 14th day of administration, showed no signs of mortality or toxicity. Moreover, acute toxicity studies showed that the median lethal dose (LD_50_) of the test sample was above 2 g kg^−1^.

### Ulcer index

Table 1 presents the results obtained in the ethanol‐induced acute GU model and shows that the UI of the ulcer control group was the highest (12.48) among all the animal groups. The UI of the CA‐C‐treated animal group was 5.23, which was nearly equal to the positive control group having a UI of 5.13. CA‐E, CA‐B, and CA‐Aq exhibited UIs of 11.75, 10.50 and 10.52 respectively. Concisely, all the fractions of *C. ambigua* showed a significant reduction in UI in comparison with the ulcer control group.

**Table 1:** Effect of *Caragana ambigua* (CA) fractions on gastric ulcer in rats

No.	Group name	Ulcer number	Ulcer score	Incidence of ulcer (%)	Ulcer index	Ulcer inhibition (%)
1	Normal control	0***	0***	0	0	100
2	Ulcer control	20.667 ± 2.76	4.1667 ± 1.18	100	12.4833	0
3	Positive (omeprazole) control	0.8333 ± 0.40***	0.5 ± 0.26***	50	5.1333	58.8785
4	CA chloroform fraction	1.6666 ± 0.92***	0.6667 ± 0.31***	50	5.2333	58.07744
5	CA *n*‐butanol fraction	3.6667 ± 0.49***	1.3333 ± 0.44**	100	10.5	15.88785
6	CA ethyl acetate fraction	15 ± 0.58***	2.5 ± 0.13^ns^	100	11.75	5.874499
7	CA aqueous fraction	3.6667 ± 0.67***	1.5 ± 0.13**	100	10.51667	15.75434

All values are given as mean plus/minus standard error of the mean. Significance: *, *P* < 0.05; **, *P* < 0.01; ***, *P* < 0.001; ns, non‐significant with respect to ulcer control group.

### Percentage of ulcer protection

The results for percentage protection against ulcer are presented in Table 1, showing values for the positive control group, CA‐C, CA‐E, CA‐B, and CA‐Aq of 58.87%, 58.07%, 5.87%, 15.88%, and 10.52% respectively. Meanwhile, Table 2 shows the gastric juice volumes, with 10.88 ± 0.33 mL being found in the ulcer control group, and 2.42 ± 0.14 mL was noted in the positive control group. The gastric juice volumes of the normal control and the CA‐C‑, CA‐E‑, CA‐B‑, and CA‐Aq‐treated animal groups were 1.45 ± 0.13 mL, 2.42 ± 0.14 mL, 3.15 ± 0.09 mL, 3.38 ± 0.12 mL, and 4.18 ± 0.08 mL respectively.

**Table 2:** Effect of *Caragana ambigua* (CA) fractions on gastric juice parameters

No.	Group name	Gastric volume (mL)	Gastric pH	Total acidity (mEq L^−1^)
1	Normal control	1.45 ± 0.13***	3.6 ± 0.06***	25 ± 2.28***
2	Ulcer control	10.883 ± 0.33	2.5333 ± 0.04	94.5 ± 3.28
3	Positive (omeprazole) control	2.417 ± 0.10***	6.0833 ± 0.04***	33 ± 2.57***
4	CA chloroform fraction	2.417 ± 0.14***	6.28 ± 0.06***	37.67 ± 3.23***
5	CA *n*‐butanol fraction	3.383 ± 0.12***	5.74 ± 0.05***	42.83 ± 6.07***
6	CA ethyl acetate fraction	3.15 ± 0.09***	5.06 ± 0.05***	56.83 ± 8.12***
7	CA aqueous fraction	4.183 ± 0.08***	4.33 ± 0.10***	61.67 ± 6.72***

All values are given as mean plus/minus standard error of the mean. Significance: *, *P* < 0.05; **,*P* < 0.01; ***, *P* < 0.001; ns, non‐significant with respect to ulcer control group.

### Estimation of gastric volume and pH

Table 2 also reports the estimation of pH for gastric juice, which was used to measure the efficiency of different fractions of *C. ambigua*. The study revealed that the normal control group has a pH of 3.6 ± 0.06 in comparison with ulcer control group exhibiting a pH of 2.53 ± 0.04. Omeprazole‑ (positive control), CA‐C‑, CA‐E‑, CA‐B_, and CA‐Aq‐treated animal groups showed pH values of 6.08 ± 0.04, 6.28 ± 0.06, 5.06 ± 0.05, 5.74 ± 0.05, and 4.33 ± 0.10 respectively.

### Estimation of total gastric acidity

Afterwards, the efficacy of the *C. ambigua* fractions was also measured in terms of acidity value (Table 2). The gastric acidity value of the ulcer control group was the highest 94.50 ± 3.28 mEq L^−1^ among all the other groups. The acidity value for the normal control group was 25 ± 2.28 mEq L^−1^, compared with 33 ± 2.57 mEq L^−1^ in the positive control (omeprazole) group. Subsequently, the acidity values of the CA‐C, CA‐E, CA‐B, and CA‐Aq groups were observed to be 37.67 ± 3.23 mEq L^−1^, 56.83 ± 8.12 mEq L^−1^, 42.83 ± 6.07 mEq L^−1^, and 61.67 ± 6.72 mEq L^−1^ respectively. The results revealed a decrease in gastric volume and total acidity and an increase in gastric pH with a significance of *P* < 0.001 for the positive control and *C. ambigua* fractions treated groups.

### Estimation of gastric wall protein and gastric mucus content

Table 3 shows the total protein content and mucus content. The total protein content in the normal control group was 474.67 ± 17.80 μg mL^−1^; this compares with a total protein value of 401 ± 31.52 μg mL^−1^ in the CA‐C‐treated. The ulcer and positive control groups have total protein values of 281.83 ± 15.30 μg mL^−1^ and 450.25 ± 31.52 μg mL^−1^ respectively, whereas the CA‐E‑, CA‐B‑, and CA‐Aq‐treated groups showed total protein values of 334.17 ± 70.09 μg mL^−1^, 395.42 ± 39.11 μg mL^−1^, and 377.40 ± 13.33 μg mL^−1^ respectively. Thus, for the protein content, the positive control group showed significant results (*P* < 0.05), whereas the outcomes of all other groups were non‐significant. The highest mucus content, of 190.58 ± 31.87 μg mL^−1^, was observed in case of CA‐C in comparison with all groups. In the ulcer control group, the mucus content was 114.92 ± 12.64 μg mL^−1^, whereas it was 177.583 ± 14.62 μg mL^−1^ in the case of the positive control. The CA‐E, CA‐B, and CA‐Aq groups exhibited mucus contents of 104.67 ± 11.25 μg mL^−1^, 112.33 ± 5.92 μg mL^−1^, and 106.25 ± 4.51 μg mL^−1^ respectively. Thus, a significant increase in mucus content was observed in CA‐C (*P* < 0.01), whereas in the positive control group it was measured to be with a significance of *P* < 0.05. All other values were non‐significant.

**Table 3:** Effect of *Caragana ambigua* (CA) fractions on protein and mucus content

No	Group name	Protein content (μg mL^−1^)	Mucus content (μg alcian blue/g wet tissue)
1	Normal control	474.667 ± 17.80**	189.583 ± 5.50**
2	Ulcer control	281.833 ± 15.30	114.917 ± 12.64
3	Positive (omeprazole) control	450.25 ± 54.47*	177.583 ± 14.62*
4	CA chloroform fraction	401.25 ± 31.52 ^ns^	190.583 ± 31.87**
5	CA *n*‐butanol fraction	395.417 ± 39.11 ^ns^	112.333 ± 5.92^ns^
6	CA ethyl acetate fraction	334.1667 ± 70.09 ^ns^	104.667 ± 11.25^ns^
7	CA aqueous fraction	377.4 ± 13.33 ^ns^	106.25 ± 4.51^ns^

All values are given as mean plus/minus standard error of the mean. Significance: *, *P* < 0.05; **, *P* < 0.01; ***, *P* < 0.001; ns, non‐significant with respect to ulcer control group.

### Macroscopic evaluation of gastric lesions

**Figure 1:**
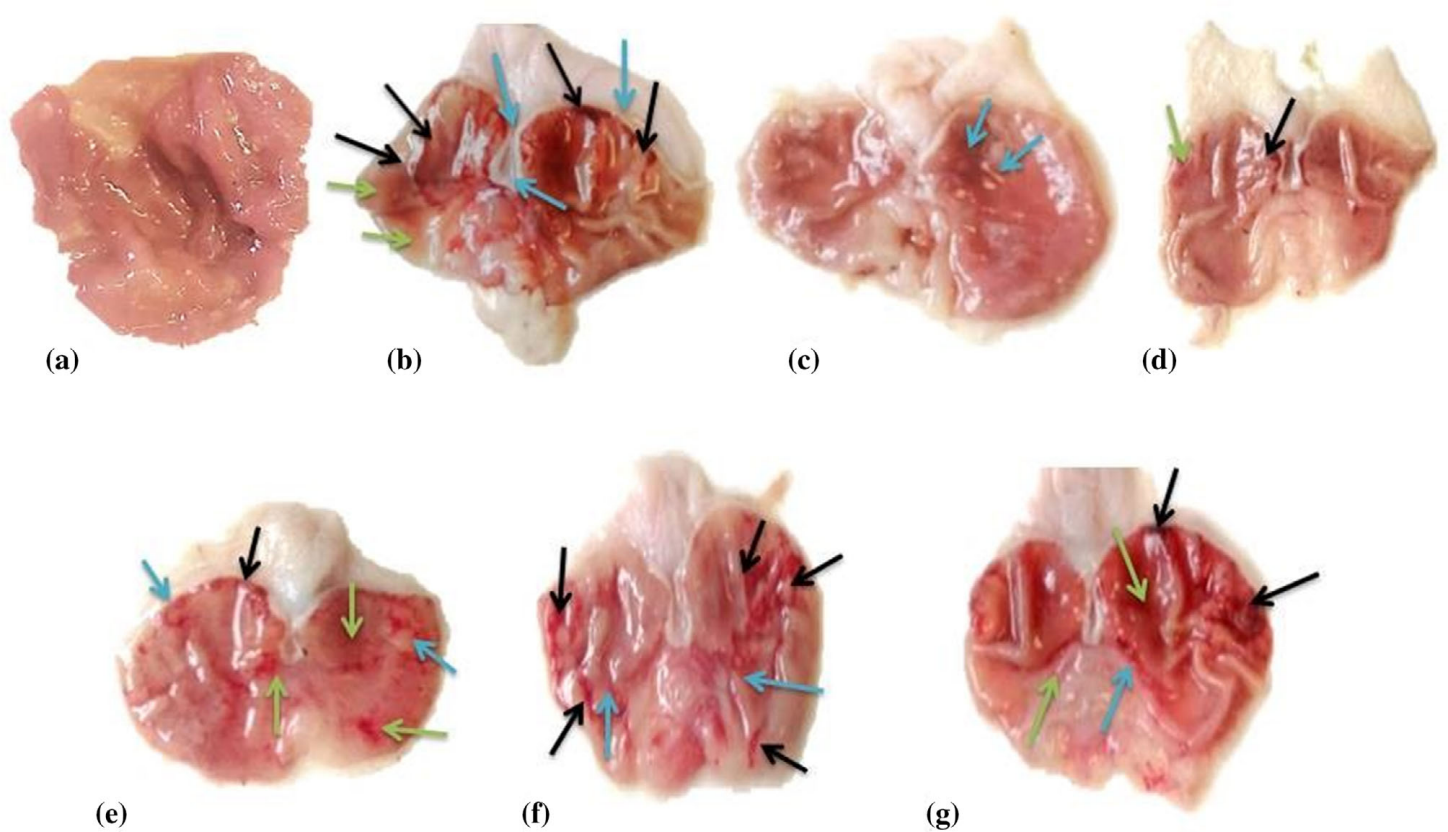
Microscopic evaluation of gastric mucosa tissue of rats: (a) normal control group; (b) disease control group; (c) positive control (omeprazole) group; (d) group treated with chloroform fraction of *Caragana ambigua*; (e) group treated with *n*‐butanol fraction of *C. ambigua*; (f) group treated with ethyl acetate fraction *C. ambigua*; (g) group treated with aqueous fraction of *C. ambigua*. Black arrows indicate hemorrhagic streaks, blue arrows indicate ulcer spots, and green arrows indicates reddish mucosa.

The macroscopic view of the gastric mucosa of normal rats showed a plane surface with no noticeable lesions. The ethanol administration induced severe damage to gastric mucosa with extensive hemorrhagic GU in rats. However, animals pretreated with omeprazole or CA‐C (300 mg kg^−1^ *per os* (p.o.)) have very mild to no disruption of gastric mucosa. Pretreatment with CA‐B (300 mg kg^−1^ p.o.) displayed mild disruption of gastric mucosa. Pretreatment with CA‐E (300 mg kg^−1^ p.o.) and CA‐Aq (300 mg kg^−1^ p.o.) represented moderate disruption of gastric mucosa, as shown in Fig. 1.

### Histological evaluation of gastric mucosa

**Figure 2:**
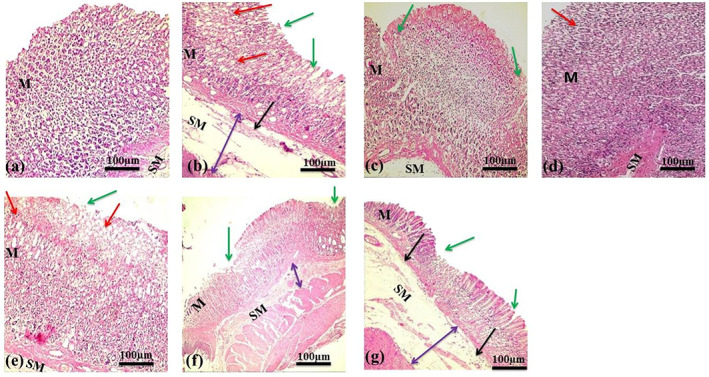
Histological evaluation of gastric mucosa tissue of rats: (a) normal control group; (b) disease control group; (c) positive (omeprazole) control group; (d) group treated with chloroform fraction of *Caragana ambigua*; (e) group treated with *n*‐butanol fraction of *C. ambigua*; (f) group treated with ethyl acetate fraction *C. ambigua*; (g) group treated with aqueous fraction of *C. ambigua*. Green arrows indicate areas of ulceration and focal erosion, red arrows indicate mucosal edema, double‐headed purple arrows indicate submucosal edema, and black arrows indicate leucocytes infiltration. M: mucosa; SM: submucosa.

The histopathological findings of stomachs of the ulcer control group revealed severe GUs that penetrated deeply into gastric mucosa. There was a wide mucosal and submucosal layer edema with leukocytes infiltration (Fig. 2(b)). The animals pretreated with omeprazole and CA‐C (300 mg kg^−1^ p.o.) presented remarkable protection of gastric mucosa compared with the disease control group. There was a decrease in mucosal injuries with no submucosal edema and leucocytes infiltration. Animal groups pretreated with CA‐B and CA‐E fractions also resulted in decreased mucosal injuries, submucosal edema, and leukocytes infiltration. CA‐Aq showed no significant protection from the damaging effect of ethanol and exhibited severe mucosal injuries, submucosal edema, and leukocytes infiltration (Fig. 2).

### UHPLC–UV–hr‐qTOF‐MS/MS

Finally, UHPLC–UV–hr‐qTOF‐MS/MS analysis of all four fractions was performed, which revealed the marked presence of isoflavones in the chloroform fraction. These compounds were absent from all other fractions analyzed. The fragmentation pattern of two of the compounds in the chloroform fraction were subjected to MetFrag *in silico* database search engine employing the ChEBI database and revealed the presence of formononetin and biochanin A. Furthermore, the fragmentation pattern was matched with the reported data in the literature^32^, ^33^, ^34^ that had confirmed the presence of formononetin and biochanin A.

## DISCUSSION

Acute toxicity findings showed that all fractions were designated safe in rats at 2000 mg kg^−1^ BW concentration, whereas the LD_50_ of fractions was greater than 2000 mg kg^−1^ BW. Therefore, in this study, a 300 mg kg^−1^ BW dose was used to evaluate the ability of *C. ambigua* to promote the protection of gastric mucosa in rats having GU.^35^ In the ethanol‐induced ulcer model, ethanol alters the gastric secretions, leading to damage of the mucosal lining and resulting in depletion of gastric mucus, disturbing the permeability and the production of free radicals.^36^ Ethanol also causes gastric lesions and hemorrhages, leading to necrosis of tissues.^37^ Gastroprotective effects of the aforementioned fractions were compared with the well‐known commercial product omeprazole. Omeprazole is an anti‐ulcer drug that exerts its effect by decreasing the amount of gastric acid production via ceasing proton pumps. It is broadly used to treat problems related to gastric acidity and protects mucosal surfaces from damage.

Extreme stomach mucosa disruption leads to the increased gastric content while bicarbonate ions release is decreased resulting inthe lowering of gastric pH.^38^ In the present study, all the *C. ambigua* fractions significantly reduced the gastric juice volume and total acidity content that resulted in increased gastric pH. *C. ambigua* fractions might exert their effect on the proton pump or histamine H_2_ receptor by acting as antagonists and, as a result, the production of acid or gastric secretions became inhibited or decreased.^39^ All the fractions of *C. ambigua* demonstrated a prominent decrease in UI in comparison with the standard drug omeprazole, as shown in Fig. 1. The percentage of ulcer protection conferred by the CA‐C fraction on the gastric mucosa was the highest and comparable to omeprazole.

Histological findings were very similar for both omeprazole (positive control, group III) and CA‐C (group IV). Both groups showed mild disruption of surface epithelium along with normal lamina propria. Moreover, no signs of edema and presence of mild leukocyte infiltration in the submucosal layer were found in the two groups. All other groups (V, VI, and VII) showed less disruption of gastric mucosa in comparison with the ulcer control group. No deep ulcers were found in all treated groups. However, shallow superficial erosions, mild to moderate edema, and leukocyte infiltration were observed in these three groups (Figs 1 and 2).

Additionally, a decrease in the amount of total protein content was marked as a sign of the impairment in normal cellular activities. Hence, treatments that cause an increase in protein content may be regarded to contain auto‐healing agents that are supportive in the process of mucosal regeneration.^40^ Low protein content in the ulcer control group was an indication of cellular dysfunction. In all experimental groups, protein content was higher compared with the ulcer control group, supporting the presence of auto‐healing agents and the gastroprotective effect of *C. ambigua* fractions. The main protective agent is the thick mucus layer that defends the stomach and plays a central role in the protection of gastric mucosa against endogenous aggressive factors, such as acid and pepsin secretion. Gastric mucus also performs a key role in promoting the restoration of ulcerative mucosa.^41^ Decreased mucus content was therefore observed in the ulcer control group. The CA‐C fraction showed the preservation of highest mucus content in comparison with the ulcer control group. Thus, the amplification of mucus production might be attributed to the ulcer healing potential of the CA‐C fraction. All other fractions showed non‐significant values regarding increase in mucus content.

The overall results lead to the proposal that *C. ambigua* has a pronounced potential to stop the progression of GU. Moreover, all the fractions of *C. ambigua* at a dose of 300 mg kg^−1^ BW displayed a great potential in treating GU. The possible mechanisms of ulcer healing potential of *C. ambigua* might be due to the ability to cease secretory actions and promote cell regeneration in ulcerative areas of gastric mucosa.

**Figure 3:**
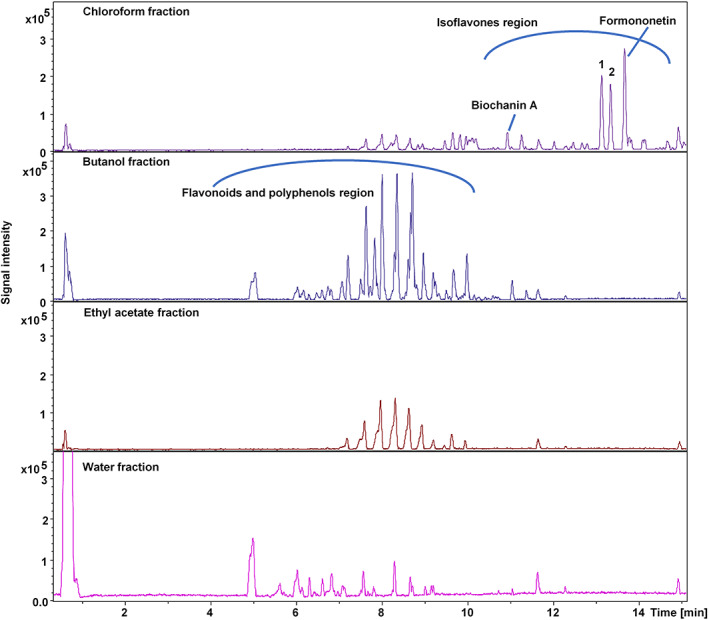
Listed base peak chromatograms of the four fractions analyzed in electrospray ionization positive mode.

UHPLC–UV–hr‐qTOF‐MS/MS data gave an insight into the chemistry of the *C. ambigua* fractions. Figure 3 shows the listed base peak chromatograms of the fractions from *C. ambigua* investigated. The compounds distinguished (absent in other fractions) in the chloroform fraction were found at retention times (RTs) of 11.0 min (biochanin A) 13.1 min (**1**), 13.4 min (**2**), and 13.7 min (formononetin) with *m*/*z* 285.073, 369.063, 283.058, and 269.079 respectively in ESI positive mode. The mass fragmentation pattern of the molecular ion M − H^+^ 269.079 (RT 13.7 min) was observed to be 254.055, 237.052, 226.060, 213.089, and 118.039, which was in accordance with the fragmentation pattern of formononetin.^33^ Furthermore, the MS^2^ transition from 269.099 to 213.089 observed in MS/MS mode strongly suggests the transition characteristic of formononetin.^34^ Therefore, the compound is characterized as formononetin. Formononetin is an isoflavonoid and also known as biochanin B. Formononetin is reported to have gastroprotective effect against ethanol‐induced gastric ulceration in rats.^42^ Moreover, alongside, formononetin, biochanin A (*m*/*z* 285.073, RT 11.0 min) was also detected in the chloroform fraction, which was confirmed through the mass fragmentation pattern 285.073, 270.050, 253.0475, 229.083, 225.052, and 213.057.^32^ Furthermore, the compound was further confirmed through MS^2^ transition of 285.073 to 253.047 in MS/MS mode and corresponds to the cited literature.^34^ Biochanin A is also an isoflavonoid that possesses anti‐ulcer potential in ethanol‐induced gastric mucosal ulcer in rats.^43^ In our opinion, the efficient anti‐ulcer properties of the chloroform fraction are due to the presence of formononetin and biochanin A. According to the best of our knowledge, this is the first instance of any scientific evidence for gastroprotective effects of *C. ambigua*. The characterization for the compounds **1** (RT 13.1 min, *m*/*z* 369.063) and **2** (RT 13.4 min, *m*/*z* 283.054) was not possible with the MS data alone and would require further nuclear magnetic resonance data. Figure 3 tentatively assigns the category of the compounds found in the different fractions of *C. ambigua* as determined through the *in silico* data analysis tool MetFrag. The CA‐C fraction of *C. ambigua* displayed the most prominent protective and anti‐ulcer effect. However, other fractions also exhibited potential to protect the gastric mucosa to some extent. Although formononetin and biochanin A were absent in CA‐E, CA‐B, and CA‐Aq fractions, these fractions also demonstrated anti‐ulcer potential to some extent. The presence of antioxidants, like polyphenols, flavonoids, tannins, and unique peptides, can be linked to the minor gastroprotective effects shown by these fractions.^14^

In conclusion, the overall results strongly suggest the positive effect of *C. ambigua* in healing GUs. Moreover, this study urges further isolation and characterization of active constituents of *C. ambigua* that are responsible for the ulcer healing and gastroprotective effect. Our findings may provide a base for the development of a new phytochemical agent that can serve as an anti‐ulcerative with minimal side effects and amplified outcomes.

## CONCLUSION

This study establishes that *C. ambigua* possesses significant potential in reducing GU progression. Although all four fractions of *C. ambigua* displayed anti‐ulcer potential to some extent, the results obtained from the chloroform fraction were the most promising. Furthermore, a comparative LC–MS/MS screening of the four stated fractions revealed the presence of formononetin and biochanin A in the chloroform fraction. Both of these secondary metabolites have reported anti‐ulcer potential. In our opinion, formononetin and biochanin A are chiefly responsible for the stated bioactivity due to the fact that these compounds were only detected in the chloroform fraction.

## DECLARATION OF INTEREST

There is no conflict of interests to declare.
